# Demonstration of Gliadin Penetration into the Epidermis by Tape Stripping as Prerequisite for Percutaneous Sensitisation in Wheat Allergy

**DOI:** 10.3390/ijms27083536

**Published:** 2026-04-15

**Authors:** Charlotte Jasmin Kiani, Valentina Faihs, Ida Folda, Claudia Kugler, Barbara Maier, Tomoharu Yokooji, Hiroaki Matsuo, Tilo Biedermann, Katharina Anne Scherf, Knut Brockow

**Affiliations:** 1 Department of Dermatology and Allergology, Technical University of Munich, 80802 Munich, Germany; charlotte.kiani@tum.de (C.J.K.); valentina.faihs@mri.tum.de (V.F.); ida.folda@tum.de (I.F.); claudia.kugler@tum.de (C.K.); tilo.biedermann@tum.de (T.B.); 2 Leibniz Institute for Food Systems Biology at the Technical University of Munich, 85354 Freising, Germany; b.maier.leibniz-lsb@tum.de (B.M.); k.scherf.leibniz-lsb@tum.de (K.A.S.); 3 Department of Frontier Science for Pharmacotherapy, Graduate School of Biomedical and Health Sciences, Hiroshima University, Hiroshima 734-8553, Japan; yokooji@hiroshima-u.ac.jp; 4 Department of Pharmaceutical Services, Hiroshima University Hospital, Hiroshima 734-8551, Japan; hmatsuo@hiroshima-u.ac.jp; 5 Food Biopolymer Systems, TUM School of Life Sciences, Technical University of Munich, 85354 Freising, Germany; 6 Odense Research Center for Anaphylaxis (ORCA), Department of Dermatology and Allergy Centre, Odense University Hospital, 5000 Odense, Denmark

**Keywords:** food allergy, WALDA, hydrolysed wheat protein, gluten, sensitisation, skin barrier

## Abstract

Sensitisation leading to food allergy may occur through the skin. Quantitative data on epidermal allergen penetration as a prerequisite for this remain limited. This study quantifies epidermal penetration of gluten and hydrolysed wheat protein (HWP) in 18 patients with challenge-confirmed WALDA (wheat allergy dependent on augmentation factors) and 12 healthy controls (HC). After 1 h of epicutaneous wheat application, 20 consecutive tape strips (TS) were collected, and wheat protein concentration was quantified by gliadin-specific ELISA. Skin barrier status was assessed by electrical impedance spectroscopy (EIS). Serum gliadin levels were measured before and 90 min after wheat application. Gliadin was detected across all TS layers for gluten and HWP. Penetration levels did not differ between patients and controls. Skin barrier status assessed by EIS did not differ significantly between individuals with and without a history of atopic dermatitis (*p* = 0.27). No correlation was observed between EIS-assessed skin barrier status and gliadin penetration. Serum gliadin was not increased after epicutaneous wheat application. This study using TS demonstrates for the first time that wheat allergens penetrated into the epidermis but could not be detected in the serum. Neither wheat allergy nor skin barrier status was associated with increased stratum corneum penetration. These findings suggest that epidermal uptake alone may not be sufficient to explain sensitisation.

## 1. Introduction

Food allergies (FAs) are on the rise, representing a growing health concern [1]. For decades, the gastrointestinal tract was considered the primary route of sensitisation in IgE-mediated FAs. However, increasing evidence indicates that the skin plays a pivotal role in the sensitisation phase [2,3,4]. The skin, known as the largest organ of the human body, serves as the primary interface against the external environment [5]. The stratum corneum (SC), the outermost layer of the epidermis, provides a highly protective barrier. Under physiological conditions, food allergens, which are mainly large molecule proteins, are not expected to penetrate the skin barrier [6,7]. However, percutaneous penetration may be facilitated when the skin barrier is impaired, as observed in patients with atopic dermatitis (AD) [2,8].

A novel subtype of WALDA (wheat allergy dependent on augmentation factors) has been described, caused by percutaneous sensitisation to hydrolysed wheat protein (HWP) in cosmetic products such as soaps [9,10,11]. WALDA is the most frequent form of wheat allergy in adults, in which additional augmentation factors (e.g., exercise and non-steroidal anti-inflammatory drugs) are required to elicit reactions upon oral wheat ingestion [11,12,13]. To date, it is unclear how this sudden, adult-onset breakdown of immunological tolerance to wheat occurs in affected individuals. Moreover, direct evidence for the penetration of wheat proteins through the skin is lacking. Further research is therefore needed to understand the mechanisms of percutaneous food allergen penetration and to identify susceptibilities contributing to sensitisation.

This study aims to quantitatively assess the percutaneous wheat protein penetration in patients with WALDA compared to healthy individuals through the SC as the upper skin barrier layer. To the best of our knowledge, this is the first human study to investigate food protein penetration using tape stripping (TS) combined with a quantitative enzyme-linked immunosorbent assay (ELISA)-based approach.

## 2. Results

### 2.1. Study Population

A total of 18 adult patients with challenge-confirmed WALDA were included in this study (median age 37 years, IQR (interquartile range) 29–56; nine female, nine male). Among them, 78% (*n* = 14) showed sIgE (specific IgE) against ω5-gliadin (Table 1). A control group of 12 tolerant healthy controls (HC) was included (median age 31 years, IQR 28–54, seven female, five male). Two individuals in the control group showed detectable sIgE against ω5-gliadin (individual 1: 0.46 kU/L; individual 2: 2.8 kU/L). In both groups, 33% reported a history of AD.

### 2.2. Evidence of Penetration into Stratum Corneum by Wheat Proteins

**Figure 1:**
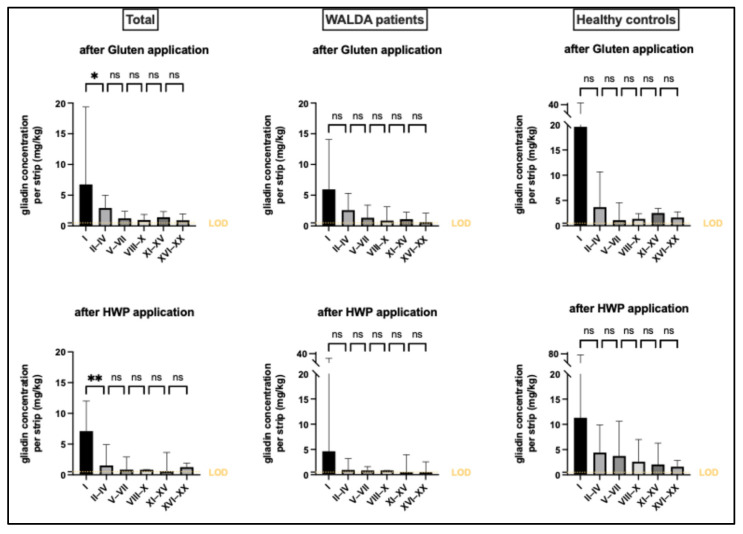
**Gliadin concentrations across the epidermal layers.** Data are presented as median (mg/kg) with interquartile range for all study participants and stratified by WALDA patients and healthy controls. The dashed line indicates the limit of detection (LOD). Statistical significance: * *p* < 0.05; ** *p* < 0.01 (Friedman test with Dunn’s post hoc test); ns = not significant.

**Figure 2:**
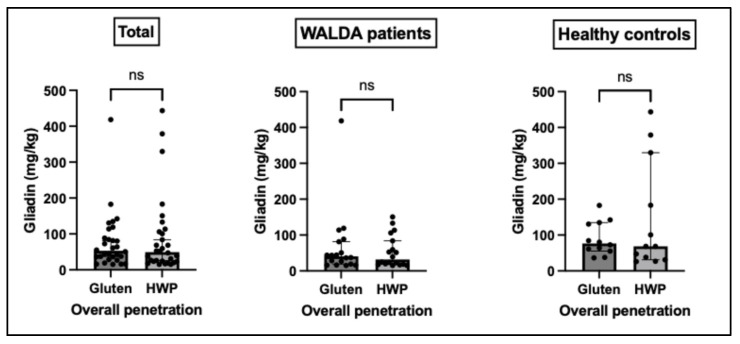
**Comparison of the overall penetration of gluten versus hydrolysed wheat protein (HWP) into the stratum corneum.** Cumulative penetration levels are shown in mg/kg for all study participants as well as stratified into WALDA patients and healthy controls. Data are presented as individual values with median and IQR (within-subject comparison; Wilcoxon matched-pairs signed-rank test); ns = not significant.

Gliadin signals were detectable across all TS layers, providing clear evidence of penetration through SC layers. Protein concentrations decreased with increasing depth. In some deeper layers, both preparations exhibited a slight, non-significant increase in gliadin signals (gluten: pooled TS fraction 4 to 5; HWP: pooled TS fraction 5 to 6). In both preparations, only layer I had significantly higher protein concentrations compared to layers II-IV (gluten *p* < 0.05, HWP *p* < 0.01; Figure 1). For both test preparations, no significant differences in penetration were found between WALDA patients and the control group across any epidermal layer (*p* > 0.05 for all layers; Figure 1). No significant differences between gluten and HWP were observed in total penetration levels, neither overall (gluten: 52.99 (34.40–94.84) vs. HWP: 49.50 (23.27–108.0); *p* = 0.43; *n* = 30) nor when stratified into WALDA patients (gluten: 40.48 (18.43–83.21) vs. HWP: 31.87 (18.35–89.46); *p* = 0.58; *n* = 18) and the control group (gluten: 76.35 (56.74–133.6) vs. HWP: 68.59 (32.93–293.1); *p* = 0.85; *n* = 12) (Figure 2). No significant interaction between skin depth and group was observed for either gluten (F(5,140) = 1.049, *p* = 0.392) or HWP (F(5,140) = 2.218, *p* = 0.056).

### 2.3. Association of AD and Barrier Impairment with Protein Penetration

**Figure 3:**
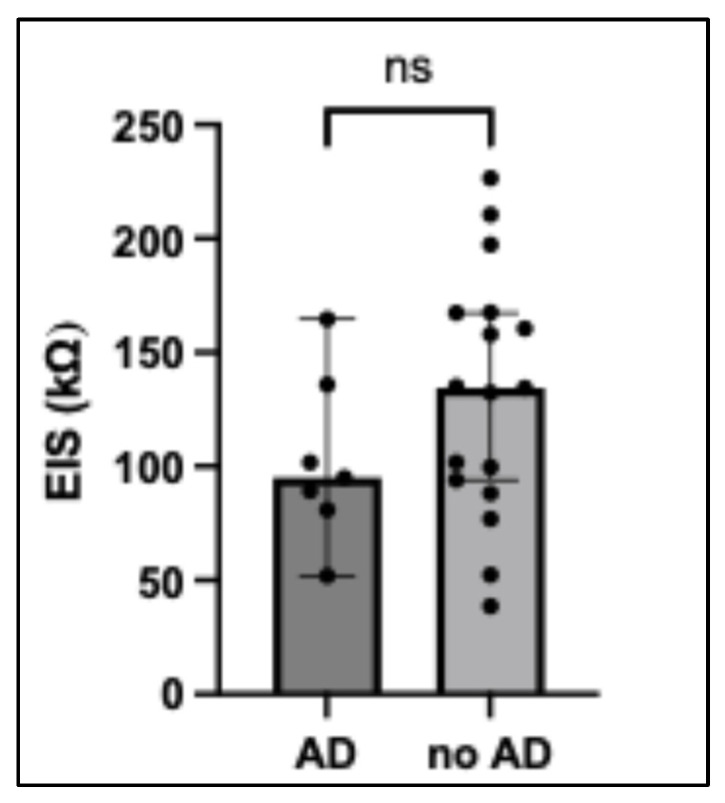
**Skin barrier function.** Assessed by electrical impedance spectroscopy in participants with a history of AD (atopic dermatitis; mild, non-active) compared to those without AD. Data are presented as individual values with median and interquartile range (Mann–Whitney U test); ns = not significant.

To address the potential influence of skin barrier impairment on skin penetration, all participants were stratified according to their history of AD (all mild and non-active). Skin barrier function was quantified by electrical impedance spectroscopy (EIS) (missing values: 6). Impedance levels in AD patients (95.1; 81.0–136.0; *n* = 7) compared to those without (134.4; 91.2–167.5; *n* = 17) were not significantly lower (*p* = 0.27; Figure 3). Spearman’s correlation analysis between skin barrier function (EIS) and gliadin concentration across the different TS depths (I, II–IV, V–VII, VIII–X, XI–XV, XVI–XX) as well as the total sum of all 20 strips did not reveal any significant associations (all *p* > 0.05).

### 2.4. Serum Gliadin Levels

**Figure A1:**
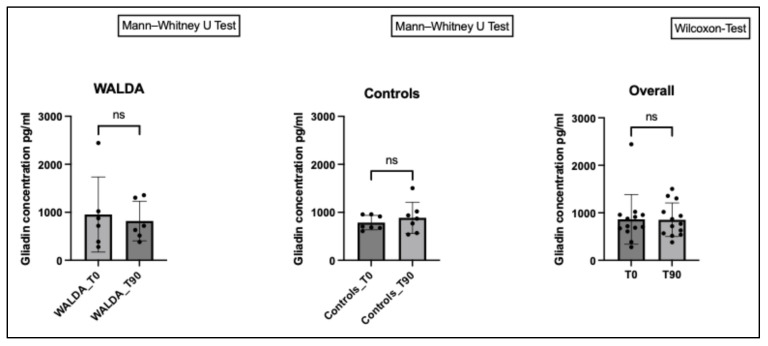
Serum gliadin concentrations across healthy controls and WALDA patients before (T0) and after (T90) tape stripping; ns = not significant.

After epicutaneous wheat protein application, no significant increase in serum gliadin levels was observed in any group compared to baseline (both groups *p* > 0.5) (see Table A1, Figure A1). No differences in serum gliadin levels between WALDA patients and controls were detected either at baseline (*p* = 0.95) or after allergen application (*p* = 0.53) (Table A1).

### 2.5. Association of Total IgE and Sensitisation Markers with Wheat Protein Penetration

Correlation analysis showed no significant association between the total epidermal penetration of gluten and total serum IgE levels (r = −0.30, *p* = 0.10). Similarly, no significant correlation was observed between the total epidermal penetration of HWP and total serum IgE levels (r = −0.19, *p* = 0.31). For ω5-gliadin sIgE, no significant correlation with total gluten penetration was observed across the total study population (r = −0.13, *p* = 0.50). No significant correlations were found after stratification into WALDA (r = 0.32, *p* = 0.20) and HC (r = −0.20, *p* = 0.55). In contrast, for HWP, total epidermal penetration in the total study population showed a moderate significant inverse correlation to ω5-gliadin-sIgE levels (r = −0.41, *p* = 0.02). The stratified analysis showed no correlation in WALDA (r = −0.05, *p* = 0.84), while significant inverse correlation was present in HC (r = −0.64, *p* = 0.03) (Table 2).

**Figure 4:**
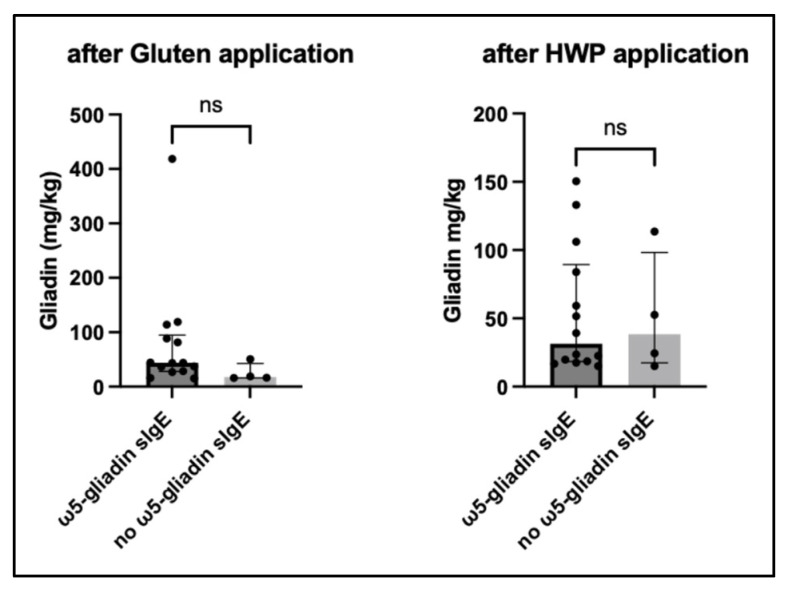
**Total epidermal penetration in WALDA patients based on ω5-gliadin sensitisation.** Data are presented as individual values with median and interquartile range (Mann–Whitney U test); ns = non-significant.

No differences in total epidermal penetration in WALDA patients with (*n* = 8) and without (*n* = 4) positive ω5-gliadin sIgE were observed. Within the WALDA patient group, the total epidermal penetration of gluten and HWP did not differ significantly between patients with detectable ω5-gliadin sIgE and those without (gluten: *p* = 0.16; HWP: *p* = 0.97) (Figure 4). Total epidermal penetration in WALDA patients did not significantly correlate with the amount of gluten and augmentation factors needed to elicit a clinical reaction in the challenge test (gluten: r = −0.39, *p* = 0.11; HWP: r = 0.05, *p* = 0.84; *n* = 18) (Table 2).

### 2.6. SDS-PAGE Characterisation of the HWP

**Figure A2:**
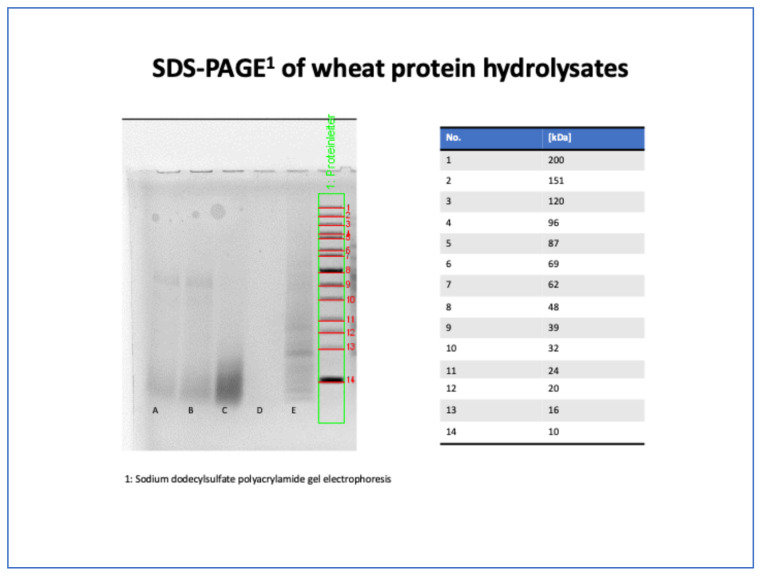
SDS-PAGE gel showing the protein pattern of different hydrolysed wheat proteins (HWPs) including the HWP used for this study (lane D). Green bar: molecular weight marker (range 10–200 kDa) with the higher molecular weight proteins located at the top of the gel; lanes A–E: analysed HWP samples.

The protein pattern of five HWPs including the one used for this study (BASF Gluadin WP) was analysed by SDS-PAGE using a mixture of proteins as a size standard (PageRulerTM Unstained Protein Ladder, Thermo Fisher Scientific, Darmstadt, Germany) ranging from 10 to 200 kDa (Figure A2). The gel showed several protein bands distributed across the different samples, indicating the presence of proteins of different molecular weight (Figure A2). Most bands appeared in the lower to middle region of the gel, suggesting the presence of smaller protein fragments. Sample D (Gluadin WP) did not show clearly distinguishable protein bands in the range of >10 kDa compared to the other samples.

## 3. Discussion

This study demonstrates by TS that food allergens can penetrate into the epidermis of intact human skin. To our knowledge, this is the first study quantifying epidermal penetration of wheat allergens into the SC using this method. Detectable gliadin signals across all TS layers indicate that epicutaneously applied wheat proteins can traverse all measured SC layers as the primary skin barrier, with decreasing concentrations towards deeper layers. The gliadin concentration of the outermost layer differed significantly from that of the adjacent layers (Figure 1). This is most likely due to a particular retention of gliadin in the outermost SC layer. However, residual surface contamination cannot be entirely excluded despite thorough cleansing, whereas removal of the first TS was generally assumed to eliminate remaining residues for the underlying layers.

The penetration of wheat proteins into the SC is surprising, as proteins with a size > 500 Dalton were originally not believed to cross the border through the SC, unless the barrier is disrupted. Wheat proteins (gliadins as well as glutenins) far exceed this threshold, with molecular weights in the kilo-Dalton range [6,7,14,15].

However, comparable results were also reported by Jacobi et al. and Baber et al., who both performed TS after epicutaneously applying either fluorescein isothiocyanate (FITC)-labelled pollen proteins (Jacobi et al.) or FITC-labelled peanut protein (Baber et al.) to healthy human skin in vivo [16,17,18]. They demonstrated the penetration of the applied proteins in decreasing concentrations into the epidermis, particularly via the hair follicles as an alternative pathway to the lipid layers [17]. Nevertheless, TS was limited to detecting gliadin within the SC and therefore does not allow conclusions about deeper epidermal penetration or transdermal systemic absorption.

Increasing evidence supports HWP in cosmetics as a cause of percutaneous sensitisation to wheat, finally leading to the development of WALDA (thus, allergic reactions to oral wheat intake in combination with augmentation factors) in some patients [9,10,19,20]. In this context, the present study aimed to assess whether WALDA patients show any predisposition to altered epidermal penetration of wheat proteins. However, no difference in protein penetration was detected between WALDA patients and HC, while acknowledging the limited sample size. This suggests that epidermal penetration may constitute an initial prerequisite for percutaneous sensitisation but is unlikely to explain it.

This study was restricted to the assessment of protein penetration alone and did not address immunological responses. Thus, a main limitation of the present study is the lack of downstream immunological outcomes, such as cellular activation and cytokine responses. Therefore, while demonstrating epidermal allergen penetration, we cannot draw conclusions regarding the mechanisms leading to sensitisation. Differences between sensitised and non-sensitised individuals may primarily arise at the level of immune recognition and costimulatory signals. The conditions of allergen exposure (e.g., amount, frequency or occlusion) are also likely to play a decisive role [8,17,18].

Jacobi et al. demonstrated that 15 min of epicutaneous allergen exposure resulted in nearly twice the allergen concentration in the upper SC compared to a 45 min application time, leading the authors to speculate that prolonged application may lead to the ongoing penetration of the pollen protein into deeper skin layers [17]. Baber et al. only incubated the applied peanut allergen for 5 min [18]. In the present study, the epicutaneous allergen application time was extended to 1 h, and wheat proteins remained detectable. Nevertheless, only a single time point was assessed, limiting potential conclusions on penetration dynamics over time. The observation of a slight increase in the protein concentration in the deeper TS layers (Figure 1) could not be confirmed in the other two studies. However, their analysis was based on a semi-quantitative approach, and methodological differences, such as variability in sensitivity and specificity of the detection method as well as differences in application time and the applied allergen itself could explain the observed discrepancies [17,18].

Furthermore, in this study, an occlusive dressing was used to ensure standardised and continuous allergen exposure to the skin. This may lead to an artificially enhanced penetration, as occlusion is known to alter the skin barrier through alterations in the corneocyte structure [8,21]. Therefore, conclusions regarding real-life exposure must be drawn carefully. Similar conditions may still occur in real-life scenarios, such as under clothing or through the application of occlusive products such as cosmetical face masks. Notably, face masks containing HWP have been previously reported to trigger WALDA [10].

In experimental murine models, it has been extensively investigated if skin barrier disruption is a susceptibility or even a prerequisite for epicutaneous sensitisation [2]. The majority of studies confirm that sensitisation only occurs in the context of barrier disruption or in the presence of exogenous adjuvants, whereas the application of food allergens (e.g., peanut or ovalbumin) onto intact skin induces immunological tolerance [22,23,24,25]. Real-life evidence supports this by showing that children with AD, a condition with an impaired skin barrier function, exhibit an increased risk for peanut sensitisation when exposed to environmental peanut protein in household dust [26].

Overall, these observations can be subsumed by the epithelial barrier hypothesis, which provides a mechanistic framework linking environmental and genetic factors to an increased risk of food allergies, while emphasising the central role of the skin barrier [2,3,27]. In this study, skin barrier status was assessed by EIS, an emerging method to assess the skin barrier function. In contrast to transepidermal water loss (TEWL), which directly measures water diffusion across the skin, EIS reflects electrical properties not only influenced by hydration, but also by structural characteristics of the SC, potentially providing additional information on barrier status. Previous studies have demonstrated a good correlation between EIS and TEWL, while suggesting that EIS may represent a robust method for assessing skin barrier status [8,28,29,30].

In this study, EIS-assessed skin barrier status was not associated with higher levels of wheat protein penetration. Although participants with a history of AD showed a trend toward reduced barrier function (Figure 3), this did not lead to increased allergen penetration. This further suggests that epicutaneous allergen uptake per se may be insufficient to explain sensitisation. However, all AD cases were mild and non-active at the time of investigation. More pronounced barrier defects, active inflammation or a genetic susceptibility such as loss-of-function mutations in the filaggrin gene may be required to facilitate percutaneous penetration. In the present study, however, the inclusion of individuals with active disease and pronounced barrier disruption was ethically not justifiable due to the potentially increased risk of inducing sensitisation. This limitation should therefore be addressed in future studies employing alternative study designs that allow the role of severe barrier disruption and active inflammation in the context of percutaneous food allergen penetration to be assessed safely.

Nevertheless, WALDA generally is not associated with an increased prevalence of atopic comorbidities, suggesting that factors other than barrier impairment may contribute to sensitisation in these individuals [11,31]. In the WALDA cases associated with the use of facial soap containing HWP, the low molecular weight and its modification through hydrolysis compared to native gluten was further discussed as a potential risk factor for percutaneous penetration and sensitisation [9,32]. In this study, wheat protein penetration into the SC occurred independently of protein type. In line with this, HWP did not enhance epidermal penetration. The SDS-PAGE analysis of the HWP did not show clearly distinguishable protein bands in the SDS-PAGE gel, suggesting that the degree of the hydrolysis of the HWP preparation was very high and the molecular weight distribution in this fraction was below the molecular weight of the lowest marker protein of 10 kDa. However, molecular size alone may be unlikely to be the primary determinant of epidermal penetration, and additional factors may be required to enable clinically relevant sensitisation. Research has shown that environmental factors such as detergents are able to impair epithelial barrier function by decreasing tight-junctions and disrupting the lamellar architecture of the SC [33,34]. Notably, the reported HWP-containing soap also contains surfactants, which may disrupt the skin barrier and thereby facilitate the epidermal uptake of wheat proteins [9]. Additionally, HWPs exhibit an altered immunoreactivity compared to native gluten due to modifications in their molecular structures [32]. This is supported by diagnostic findings, as sIgE levels to ω5-gliadin, the major allergen in conventional WALDA, are typically lower in patients with HWP-WALDA [9,11]. Interestingly, in this study, for HWP, the total epidermal penetration showed a significant inverse correlation to ω5-gliadin sIgE levels. Possible explanations could include faster translocation of HWP through the epidermis or more efficient capture by Langerhans cells, leading to reduced epidermal retention. However, when stratified by group, this association was only observed in HC and not in WALDA patients. Therefore, the biological relevance of this finding remains uncertain. Since these analyses are exploratory in nature, no formal correction for multiple testing was applied, and interpretation of the findings should remain cautious.

Among WALDA patients, sIgE to ω5-gliadin was not significantly associated with total epidermal penetration, although the analysis was limited by the small number of ω5-gliadin-negative WALDA patients (*n* = 4).

Food allergen penetration through the skin is considered both a potential route for the development of food allergy and a therapeutic target. Epicutaneous immunotherapy (EPIT) represents a novel approach in allergen-specific immunotherapy. It targets the epidermis, where food allergens are taken up by Langerhans cells for local antigen presentation. A major advantage of EPIT is the limited systemic allergen distribution, which may result in a lower risk of severe allergic reaction compared to other routes of immunotherapy [35,36,37]. In line with this, the present study demonstrated that no significant increase in serum gliadin levels was observed after epicutaneous allergen application, supporting the assumption of predominantly local allergen exposure. Previous studies investigating oral gluten exposure have reported that gliadin is detectable in serum within 30 min after ingestion with a plateau reached after about 60–90 min [12,38] Although these data relate to gastrointestinal uptake, they served as a general orientation when selecting the sampling time point.

However, the results of clinical EPIT trials indicate that the risk of systemic allergic side effects is closely related to the degree of SC disruption, again underscoring the critical role of skin barrier integrity for the efficacy of epicutaneous allergen delivery [37].

A major strength of this study is the first-time use of a quantitative methodology to assess the penetration of clinically relevant wheat allergens in vivo. However, complementary methods such as immunohistochemistry could have provided additional spatial resolution regarding the exact depth of allergen localisation and should be considered in future studies as well as the inclusion of downstream immunological parameters. Also, the relatively small sample size may limit the ability to detect smaller group differences. In addition, the absence of a negative control (e.g., occlusion-only or vehicle-only condition) and its background signal analysis in the ELISA may limit the generalizability of the findings. Furthermore, penetration under non-occlusive conditions may differ, thus future studies comparing occlusive to non-occlusive exposure are needed to better reflect all possible real-life exposure scenarios.

## 4. Materials and Methods

### 4.1. Study Population and Data Collection

A total of 18 adult patients with challenge-confirmed WALDA participated in this study between January 2023 and December 2024. Patients were eligible if they had a history of allergic reaction to wheat ingestion in combination with augmentation factors, while showing tolerance to wheat consumption in conventional amounts or augmentation factors alone, and a positive OCT (oral challenge test) to wheat gluten alone at high doses or with augmentation factors. Additionally, a control group of 12 tolerant individuals, demonstrated by negative OCT with wheat gluten in combination with augmentation factors, was included. With an assumption of 80% statistical power, the present sample size (18 WALDA patients and 12 controls) would allow the detection of large effect sizes d = 1.1, while smaller effects may not have been detectable. All participants were advised to adhere to a wheat-free diet 24 h prior to the study and gave their written informed consent prior to participation in the study. The study received approval from the ethics committee of the Technical University of Munich (approval number 477/21 S-NP).

### 4.2. Demographical Data and Allergy Diagnostics

Demographic information was collected from all patients and control subjects. Blood samples were obtained to measure routine allergy diagnostics including total IgE levels and sIgE levels against wheat, gluten, gliadin, and ω5-gliadin using the ImmunoCAP assay (Thermo Fisher Scientific, Waltham, MA, USA). OCTs were performed according to a previously published protocol using increasing doses of wheat gluten and the sequential addition of augmentation factors [11]. An OCT was deemed positive upon development of objective allergic reactions, at which point the test was immediately stopped and antiallergic therapy was administered. Reaction thresholds were assessed using an ordinal scale from 1 to 10, as previously published [11].

### 4.3. Experimental Setup

#### 4.3.1. Skin Barrier Analysis

Skin barrier was quantified by EIS with the Nevisense^®^ device (SciBase AB, Stockholm, Sweden), measuring skin impedance at 35 frequencies ranging from 1 kHz to 2.5 MHz, across 4 depths and 10 permutations [8,28,29,30]. According to the manufacturer’s instructions, prior to each measurement, the skin was moistened with a wound cleansing wipe (Salvequick^®^ wound cleansing wipe, Orkla Wound Care, Solna, Sweden). By retracting the outer sleeve of the device for 10 s, the measurement was then performed. For analysis, the Z1 value (kΩ) was extracted post hoc, representing the mean value of 10 permutations at 1 kHz.

#### 4.3.2. Wheat Application and Tape Stripping

**Figure 5:**
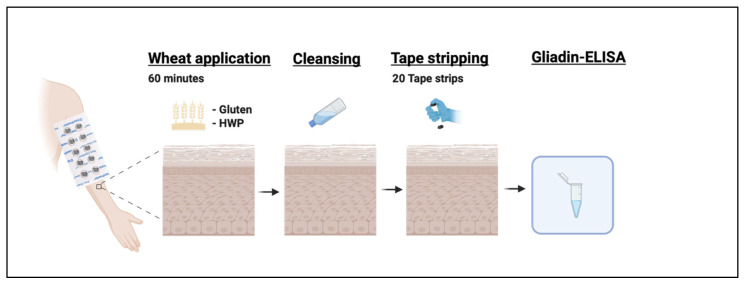
**Study protocol.** Created in BioRender. Köberle, M. (2026). https://BioRender.com/v2qhup9.

Two different wheat preparations were used, containing either native gluten flour (Vital wheat gluten, Kröner-Stärke GmbH, Ibbenbüren, Germany) or HWP (Gluadin^®^ WP, BASF SE, Ludwigshafen, Germany), both dissolved in petrolatum (Vaseline^®^, Bombastus-Werke AG, Freital, Germany) in a concentration of 15.6%. Two square areas of 6.25 cm^2^ were marked on the same volar clinically healthy forearm. A defined amount of test preparation (25 mg/cm^2^) was applied under transparent occlusive dressing. After 60 min, test preparations were carefully removed by swiping with a damp cloth from the skin to avoid contamination, as recommended by Grégoire et al. [39]. Subsequently, the TS area was marked and 20 consecutive TS (D-Squame^®^, CuDerm, Dallas, TX, USA) were applied, as previously described in detail [8]. An overview of the study protocol is shown in Figure 5.

#### 4.3.3. Sample Preparation and Sample Analysis

For further analysis, samples were pooled according to layer depth to increase analytical sensitivity, as described in previous TS studies [40,41]. Smaller pools were used for superficial strips and larger pools for deeper layers, expecting a layer-dependent decrease in concentration.

The first strip was analysed separately to exclude potential contamination, whereas strips II–IV, V–VII, VIII–X, XI–XV, and XVI–XX were pooled, resulting in five different depths per participant. For each layer, the mean gliadin content per strip was calculated. The pooled TS were furthermore eluted in 60% ethanol in an ultrasonic bath for 15 min. The resulting eluates were stored at −20 °C until analysis.

For the final analysis, gliadin concentration in the TS eluates was assessed using the Ridascreen^®^ Gliadin R7001 sandwich enzyme-linked immunosorbent assay (ELISA) (R-Biopharm AG, Darmstadt, Germany), according to the manufacturer’s instructions, with a detection limit of 0.5 mg/kg (ppm).

#### 4.3.4. Gliadin Detection in Serum

Blood samples were additionally collected before (T0) and 90 min after (T90) application of the wheat preparation. T90 was chosen to assess potential systemic gliadin levels at the end of the experimental procedure. Analysis of the serum gliadin levels was performed using a sandwich enzyme-linked immunosorbent assay kit (Morinaga Institute of Biological Science, Yokohama, Japan), with an additional vacuum centrifugation step as previously described in detail [42]. The assay has a lower detection limit of 15 pg/mL.

#### 4.3.5. SDS-PAGE of the HWP

The Sodium Dodecyl Sulfate-Polyacrylamide Gel Electrophoresis (SDS-PAGE) was performed as described in Gabler et al. [32]. Briefly, the samples were analysed on a homogenous NuPAGE 10% polyacrylamide Bis-Tris gel (10 × 1 mm wells) (Invitrogen, Carlsbad, CA, USA) using a mixture of proteins as a size standard (PageRulerTM Unstained Protein Ladder, Thermo Fisher Scientific, Waltham, MA, USA). The samples were mixed with 1 mL of extraction buffer (200 g/L sucrose, 59.7 g/L Tris-HCl, 40 g/L SDS, 0.3 g/L EDTA, 0.4 g/L Coomassie blue, 0.1 g/L phenol red, 0.11 mmol/L HCl) containing 7.72 g/L DTT, incubated for 12 h, heated to 60 °C for 10 min and centrifuged (5 min, 5000× *g*, 20 °C). The MOPS running buffer used contained 20.9 g/L 3-(N-morpholino) propane sulfonic acid (MOPS), 12.1 g/L Tris-HCl, 2 g/L SDS, 0.6 g/L EDTA, and 0.77 g/L DTT as the reducing agent. The experiment was conducted for a duration of 30 min at an input current of 115 mA and an applied voltage of 200 V. The protein bands were subjected to fixation with 12% (*w*/*w*) trichloroacetic acid for a period of 30 min, followed by staining with Coomassie blue for a further 30 min. Thereafter, destaining was initiated using a mixture of methanol, glacial acetic acid, and water (in a volume ratio of 50:10:40), followed by a second destaining step with a methanol-based solution composed of glacial acetic acid and water (in a volume ratio of 10:10:80).

The gels were subjected to scanning using the Gel DocTM EZ Imager (Bio-Rad Laboratories, Munich, Germany) and the Image Lab software (Bio-Rad Laboratories, Munich, Germany; version 6.1).

### 4.4. Statistical Analysis

Statistical analysis and data visualisation were performed using SPSS (IBM Corp., Armonk, NY, USA; version 29.0) and GraphPad Prism (GraphPad Software, Boston, MA, USA, version 10). Continuous variables are presented as median and IQR. Categorical variables are shown as frequencies and proportions. The Shapiro–Wilk test was used to assess the normality of data distribution. Only statistical analysis for non-normally distributed data was applied. The Mann–Whitney U test was applied to evaluate group differences between WALDA patients and HC. The Wilcoxon signed-rank test was used to evaluate within-group changes. The Friedman test followed by Dunn’s post hoc test was applied to analyse differences across repeated measures. Spearman’s rank correlation coefficient was used to assess correlations, without correction for multiple testing. To assess whether penetration profiles differed between groups, a linear mixed-effects model (REML) with a repeated measures design was applied to assess differences in penetration profiles between groups. A two-sided *p*-value of <0.05 was set as statistically significant. Accordingly, *p*-values ≥ 0.05 were interpreted as statistically not significant.

## 5. Conclusions

To summarise, our findings indicate that wheat proteins can penetrate into the SC, even in the absence of barrier impairment. Allergen penetration in WALDA patients was not increased compared to HC, suggesting no detectable association between epidermal allergen penetration and disease status in this cohort. These findings highlight that epidermal penetration alone may not be sufficient to explain sensitisation in these patients. Sensitisation represents a complex immunological process that extends beyond allergen penetration. The findings of this study may serve as a foundation for larger cohort studies as well as investigations into immunological mechanisms of percutaneous sensitisation. The findings underline the need for research on allergen distribution in the deeper skin layers, where an interaction with the cutaneous immune system occurs. Immunological markers and cellular interactions should be studied in more detail, for instance in suitable mechanistic animal or ex vivo human skin models, to better understand the mechanisms underlying percutaneous sensitisation and wheat allergy/WALDA development.

## Figures and Tables

**Table 1:** Clinical characteristics of WALDA patients and healthy controls.

Clinical Patient Characteristics	WALDA Patients, *n* = 18 (Percentage/Range)	Healthy Controls, *n* = 12	*p*-Value
Age, median (IQR)	37 years (29–56)	31 years (28–54)	ns
Sex, male/female, *n* (%)	9/9 (50%/50%)	5/7 (42%/58%)	ns
Any self-reported atopic comorbidities, *n* (%)	10 (56%)	7 (58%)	ns
Self-reported atopic dermatitis, *n* (%)	6 (33%)	4 (33%)	ns
Total IgE, median (IQR)	182.5 kU/L (107.5–282.0)	37.0 kU/L (5.2–377.0)	<0.05
sIgE to ω5-gliadin, median (IQR)	7.40 kU/L (0.97–8.83)	<0.1 kU/L (<0.1–2.8)	<0.001
sIgE to wheat, median (IQR)	0.29 kU/L (0.10–0.78)	<0.1 kU/L (all)	ns
sIgE to gluten, median (IQR)	1.07 kU/L (0.37–1.81)	<0.1 kU/L (<0.1–0.21)	<0.001
sIgE to gliadins, median (IQR)	0.63 kU/L (0.11–1.44)	<0.1 kU/L (<0.1–0.05)	<0.001
sIgE to rTri a 14 wheat, median (IQR)	<0.1 kU/L (<0.01–0.1)	<0.1 kU/L (all)	ns
Oral challenge test reaction threshold *, *n* (%)	(1) 4 (22.2%) (2) 1 (5.6%) (3) 2 (11.1%) (4) 5 (27.8%) (5) 0 (6) 1 (5.6%) (7) 1 (5.6%) (8) 4 (22.2%) (9) 0 (10) 0	/	/

* See previous publication for detailed description of the oral food challenge protocol and challenge steps [11]; ns = non-significant.

**Table 2:** Correlation between wheat protein penetration and IgE markers.

Correlation Parameter	Population	r (Spearman)	*p*-Value
Gluten penetration vs. Total IgE	Total study population	−0.30	ns
HWP penetration vs. Total IgE	Total study population	−0.19	ns
Gluten penetration vs. ω5-gliadin sIgE	Total study population	−0.13	ns
Gluten penetration vs. ω5-gliadin sIgE	WALDA patients	0.32	ns
Gluten penetration vs. ω5-gliadin sIgE	HC	−0.20	ns
**HWP penetration vs. ω5-gliadin sIgE**	**Total study population**	**−0.41**	**0.02**
HWP penetration vs. ω5-gliadin sIgE	WALDA patients	−0.05	ns
**HWP penetration vs. ω5-gliadin sIgE**	**HC**	**−0.64**	**0.03**
Gluten penetration vs. OCT threshold	WALDA patients	−0.39	ns
HWP penetration vs. OCT threshold	WALDA patients	0.05	ns

ns = not significant.

## Data Availability

The data that support the findings of this study are available from the corresponding author upon reasonable request.

## Abbreviations

The following abbreviations are used in this manuscript:

HWP	Hydrolysed wheat protein
WALDA	Wheat allergy dependent on augmentation factors
HC	Healthy controls
TS	Tape stripping
EIS	Electrical impedance spectroscopy
ns	Not significant
SC	Stratum corneum
ICR	Interquartile range
sIgE	Specific IgE
kU	Kilo units
EPIT	Epicutaneous immunotherapy
OCT	Oral challenge test
AD	Atopic dermatitis
kΩ	Kilo ohm
FITC	Fluorescein isothiocyanate

## Appendix A

**Table A1:** Serum gliadin levels at T0, T90 and ΔT90–T0 in pg/mL (median and interquartile range).

Condition	Total, *n* = 13	WALDA Patients, *n* = 6	Healthy Controls, *n* = 7	*p*-Value (Group Comparison)
T0	718.9 (641.1–957.3)	797.3 (358.9–1377)	703.4 (674.1–956.5)	ns (0.95)
T90	854.6 (554.0–1161.0)	675.3 (483.4–1317.0)	868.3 (569.3–1019.0)	ns (0.53)
ΔT90–T0	−3.56 (−245.8–386.3)	116.9 (−679.9–480.1)	−23.09 (−188.7–344.8)	ns (>0.99)
